# Deficiency of peripheral CLA^+^ Tregs and clinical relevance in Behcet’s syndrome

**DOI:** 10.1186/s13075-024-03306-9

**Published:** 2024-03-21

**Authors:** Jiachen Li, Feng Sun, Danxue Zhu, Yuke Hou, Gong Cheng, Ping Wang, Xu Jin, Wenyan Zhou, Xiaolin Sun, Zhanguo Li, Tian Liu

**Affiliations:** 1 Department of Rheumatology and Immunology, PKUPH, Beijing, 100044 China; 2 Beijing Key Laboratory for Rheumatism Mechanism and Immune Diagnosis (BZ0135), Beijing, 100044 China; 3 Shijiazhuang People’s Hospital, Shijiazhuang, 050030 China

**Keywords:** Behcet’s syndrome, Flow cytometry, CLA^+^ Tregs, Arterial aneurysms

## Abstract

**Background:**

Autoimmune responses have been suggested to involvement in patients with Behcet’s syndrome (BS). There has been growing attention towards the roles of cutaneous lymphocyte antigen (CLA)^+^ regular T cells (Tregs) in autoimmune diseases. The role of CLA^+^ Tregs in BS is still uncertain. This study aims to clarify the impact of CLA^+^ Tregs on BS.

**Methods:**

We collected peripheral blood from a total of 107 patients with BS and 114 healthy controls (HCs). The number of CLA^+^ Tregs, natural killer (NK) cells, B cells, and several subtypes of CD4^+^ T cells were detected using flow cytometry and compared between patients and HCs.

**Results:**

The absolute number and proportion of CLA^+^ Tregs among CD4^+^ T lymphocytes and CD4^+^ Tregs were lower in patients with BS than in HCs. CLA^+^ Tregs were positively related with NK cells (*r* = 0.500, *P* < 0.001) and B cells (*r* = 0.470, *P* < 0.001) and negatively related with effector T cells (*r*=-0.402, *P* < 0.001) in patients with BS. Patients with BS and arterial aneurysms had CLA^+^ Treg cell deficiency. A decreased proportion of CLA^+^ Tregs was associated with arterial aneurysms in patients with BS. The proportion of CLA^+^ Tregs in patients with BS increased with corticosteroids and immunosuppressants.

**Conclusion:**

CLA^+^ Tregs decrease in association with arterial aneurysm in patients with BS. CLA^+^ Tregs may be a predictor of response to BS treatment.

**Supplementary Information:**

The online version contains supplementary material available at 10.1186/s13075-024-03306-9.

## Background

Behcet’s syndrome (BS) is an autoimmune disease characterized by recurrent oral ulcers, genital ulcers, uveitis, arthritis, and nervous system lesions [1]. The pathological basis of BS is systemic vasculitis, which may invade large, medium, or small vessels throughout the body. BS mainly affects people younger than 40 years of age and rarely develops in elderly patients. BS has an increased incidence and prevalence in the Middle East and Far East Asia, where it is called Silk Route disease. The prevalence of BS was approximately 14.0 per 100,000 individuals in China by the year of 2021 [2]. 

Despite several studies investigating the pathogenesis of BS, the complete mechanism underlying this autoimmune disease remains unknown. It is recognized as an autoinflammatory disorder that occurs in individuals with a genetic predisposition, as previous research has demonstrated the presence of certain alleles, such as HLA-B51, in a subset of patients with BS [3]. Environmental factors, including infection with specific pathogens, are believed to perturb the immune systems of susceptible individuals, triggering the onset of BS [3, 4]. Dysregulated T cells play a crucial role in the immunological abnormalities observed in patients with BS. Recent studies have revealed that distinct T lymphocyte subpopulations can interact with various cytokines, leading to an inflammatory response in patients with BS [2, 5]. A disturbed Th1/Th2 balance, increased levels of Th17, and decreased levels of regular T cells (Tregs) have been reported in patients with BS. Inflammatory factors such as TNF-α, IFN-γ, IL-1, and IL-17 also contribute to the immunopathogenesis of BS. Neutrophils, natural killer (NK) cells, macrophages, and dendritic cells are believed to play important roles in BS, leading to systemic immune abnormalities [6, 7]. 

Cutaneous lymphocyte antigen (CLA) is a cell surface marker mainly expressed on T cells and is a specific homing receptor for immune cells. Previous studies have demonstrated that CLA^+^ T cells are associated with several neoplastic and non-neoplastic skin diseases [8, 9]. Specifically, CLA^+^ Tregs are a subset of Tregs that are involved in maintaining the balance of immunity [10]. The role of immune cell subpopulations, including CLA^+^ Tregs, in autoimmune diseases has attracted increasing attention in recent years; however, the effects of CLA^+^ Tregs on BS remain unclear. Therefore, this real-world study explores the changes of CLA^+^ Tregs and the association between CLA^+^ Tregs and different organ involvement in BS.

## Methods

### Patients

This retrospective, single-centre study included patients diagnosed with BS at the Department of Rheumatology and Immunology, Peking University People’s Hospital, Beijing, China between 2018 and 2022. All patients met the 2014 International Criteria for Behcet’s Disease and were newly diagnosed with BS. Patients overlapping with other autoimmune diseases (such as rheumatoid arthritis, Sjogren syndrome) were excluded. Additionally, a total of 114 individuals in good health were recruited for the study, creating a control group.

### Clinical features

The age, disease duration, previous medications, and various manifestations (fever, oral ulcers, genital ulcers, uveitis, skin lesions, gastrointestinal involvement, vasculopathy, arthritis, and nervous system lesions) of patients with BS were recorded during their initial clinical visit. Inflammatory indices, including the erythrocyte sedimentation rate (ESR) and C-reactive protein (CRP) level, were also noted. The Behcet’s Disease Current Activity Form (BDCAF) was used to evaluate disease activity [11]. 

### Flow cytometry analysis

A single-platform flow cytometry-based absolute count technique was used to quantify percentage and number of each lymphocyte subset. Add 50 µl of EDTA anticoagulated venous blood to the BD Trucount™ tube (BD, #340,334) by reverse pipetting. Cells were stained for 15 min with 10 µl anti-CD3-FITC/CD8-PE/CD45-PercP/CD4-APC antibody and CD3-FITC/CD16 + CD56-PE/CD45-PerCP/CD19-APC antibody in BD Multitest™ IMK kit (BD, #662965) without contact with blood. Then, cells are mixed with 450 µl of 1X BD Multitest IMK kit lysing solution (BD, #349202) for 15 min and detected by MultiSET software on a BD FACSCanto flow cytometer (BD Biosciences, San Jose, CA) within 24 h.

Fresh collected whole blood was processed within 24 h. To determine the proportions of T cell subsets in the periphery, immunophenotyping of T cells was classified using conjugated anti-human murine monoclonal antibodies (mAbs) as follows: anti-CD3-APC-H7, anti-CD4-FITC, anti-CD25-PE, anti- CD127 (IL-7Rα)-BV605, anti-CD45RA-BV510, anti-CD197 (CCR7)-BV421, anti-PD-1-PE-CY7, anti-human CD185 (CXCR5)- AF647, anti-CD161-BV421, anti-CLA-AF647, anti-CD3-PERCP. All antibodies were purchased from BD Pharmingen (San Diego, CA). For surface cell staining, incubate 100 µl whole blood with antibody for 15 min at room temperature in the dark. Then, 2 mL of 1X BD Multitest IMK kit lysing solution is added for 10 min for erythrocyte lysis. Thereafter, centrifuge the cells at 600 g for 5 min and remove the supernatant. Stained cells are washed with phosphate-buffered saline (PBS) and all samples are kept cool and dark until flow analysis. After appropriate instrument setup, the labeled T cell subsets were collected on a FACSAria II flow cytometer (BD Biosciences, San Jose, CA) and each T cell subset was analysed using FlowJov10 (TreeStar, Woodburn, OR).

In terms of absolute numbers, lymphocytes were gated by CD45/side scatter dot plots along with T lymphocytes (CD3^+^), B lymphocytes (CD19^+^), helper/inducer T lymphocytes (CD3^+^CD4^+^), suppressor/cytotoxic T lymphocytes (CD3^+^CD8^+^), and natural killer (NK) lymphocytes (CD3^–^CD16^+^ and/or CD56^+^). All the lymphocyte population was detected on the basis of forward scatter (FSC) and side scatter (SSC) characteristics. CLA^+^ Tregs were defined as CD3^+^CD4^+^CD25hiCD127lowCLA^+^.

### Follow up

We longitudinally traced the level of CLA^+^ Treg cells of 12 BS patients in this cohort. In these patients, the median follow-up time was 11.00 (6.25, 15.75) months. We recorded the proportion of CLA^+^ Tregs among CD4^+^ T cells and clinical manifestation before and after treatment with corticosteroids or immunosuppressants.

### Statistical analysis

All statistical analyses were performed using SPSS software (version 23.0; SPSS Inc., Chicago, IL, USA) and Prism 8 software (GraphPad Software, San Diego, CA, USA). Student’s t-test was utilized to compare continuous variables with a normal distribution between the groups. The Wilcoxon rank-sum test was used to compare continuous variables without normal distribution. Continuous variables are expressed as mean and standard deviation or median (interquartile range). Spearman linear correlation analysis was used to analyse the association between CLA^+^ Tregs and NK cells, effector T cells and B cells. Risk factors were assessed using logistic regression analysis. Statistical significance was set at *P* < 0.05.

## Results

### Patient characteristics

A total of 107 patients (55.1% males) with BS were included in this study. The median patient age was 40.0 years (interquartile range: 32.0–52.75 years), and the median disease duration was 84.0 months (interquartile range: 24.0–180.0 months). The median BDCAF score at the first clinical visit was 3.0 (interquartile range: 2.0–4.0) and all patients were in active BS (BDCAF≥1.0). Recurrent oral aphthae occurred in 102 (95.3%) patients, and 58 (54.2%) presented with genital ulcers. Other clinical manifestations included pseudofolliculitis (*n* = 44; 41.1%), erythema nodosum (*n* = 31; 29.0%), arthritis (*n* = 26; 24.3%), uveitis (*n* = 25; 23.4%), gastrointestinal involvement (*n* = 23; 21.5%), neurological involvement [*n* = 20; 18.7%, including parenchymal (*n* = 15; 14.0%) and nonparenchymal (*n* = 5; 4.7%) neuropathy], arterial aneurysm (*n* = 15; 14.0%), fever (*n* = 13; 12.1%) and thromboembolism (*n* = 12; 11.2%). Gastrointestinal involvement includes intestinal ulcers, recurrent diarrhoea and haematochezia. The patients’ characteristic and treatment were listed in Table 1. The patients with BS were divided into 5 groups according to the previous medications. [Treatment naïve group (*n* = 58; 53.2%), treated with glucocorticoids alone group (*n* = 11; 10.1%), treated with immunosuppressants/immunomodulators group (*n* = 15; 13.8%), treated with glucocorticoids and immunosuppressants/immunomodulators group (*n* = 19; 17.4%) and treated with biological agents group (*n* = 4; 3.7%)] Wilcoxon rank-sum test demonstrated that the absolute number of CLA^+^ Tregs in treated with GC alone group was significantly higher than that in treated with GC + IS/IM group and no other significant difference was found between groups. (Figure S1-S3)

**Table 1:** Patients’ characteristic

Characteristic	Data
**Age (year)**	40.0(32.0, 52.75)
**Sex**	
Male	59 (55.1%)
Female	48 (44.9%)
**Disease duration (month)**	84.0 (24.0–180.0)
**BDCAF**	3.0 (2.0, 4.0)
**Symptom**	
oral aphthae	102 (95.3%)
genital ulcers	58 (54.2%)
pseudofolliculitis	44 (41.1%)
erythema nodosum	31 (29.0%)
arthritis	26 (24.3%)
uveitis	25 (23.4%)
gastrointestinal involvement	23 (21.5%)
neurological involvement	20 (18.7%)
parenchymal	15 (14.0%)
nonparenchymal	5 (4.7%)
arterial aneurysm	15 (14.0%)
fever	13 (12.1%)
thromboembolism	12 (11.2%)
**Previous medications**	
glucocorticoids	30 (27.5%)
thalidomide	28 (26.2%)
colchicine	14 (13.1%)
azathioprine	8 (7.5%)
hydroxychloroquine	6 (5.6%)
mycophenolate mofetil	4 (3.7%)
calcineurin inhibitor	4 (3.7%)
biological agents	4 (3.7%)
cyclophosphamide	2 (1.9%)

BDCAF, Behcet’s disease current activity form score

### CLA^+^ Tregs in patients with BS

**Fig. 1:**
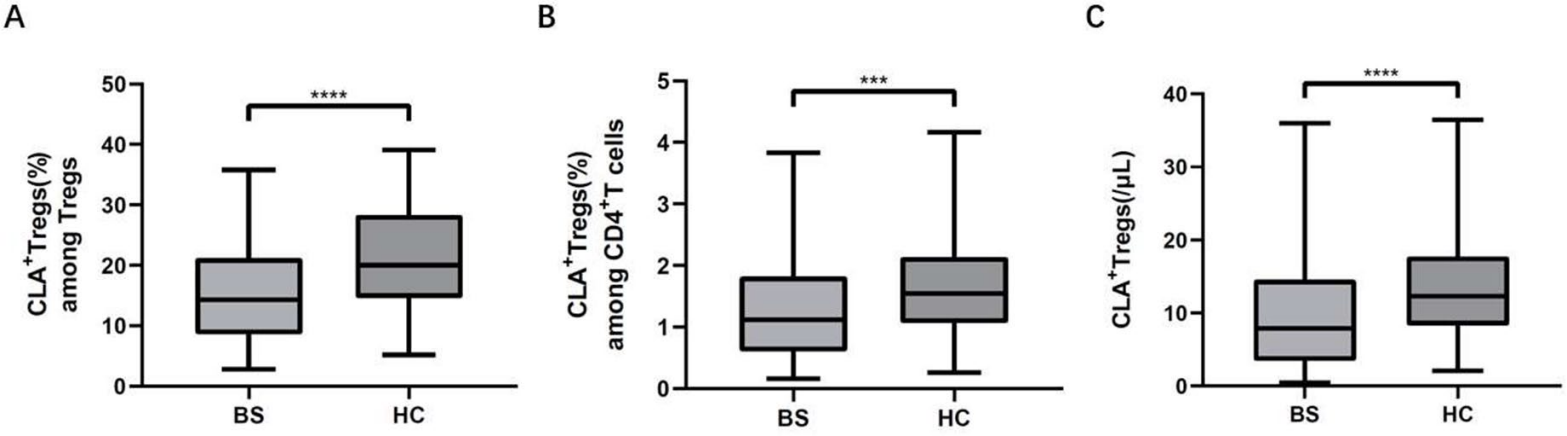
CLA^+^ Tregs (**A**) Patients with BS had a lower proportion of CLA^+^ Tregs among CD4^+^ T cells than healthy individuals(*P* < 0.05). (**B**) Patients with BS had a lower proportion of CLA^+^ Tregs among CD4^+^ T cells than healthy individuals(*P* < 0.05). (**C**) The absolute number of CLA^+^ Tregs was lower in patients with BS than in healthy individuals(*P* < 0.05). BS Behcet’s syndrome, HC Healthy controls. ***P < 0.01. ****P < 0.001

The proportions of CLA^+^ Tregs among Tregs (14.65% [interquartile range: 8.93–21.63%] vs. 20.55% [interquartile range: 14.60–28.43%], *P* < 0.001) and among CD4^+^ T lymphocytes (1.18% [interquartile range: 0.61–1.91%] vs. 1.61% [interquartile range: 1.78–2.15%], *P* = 0.001) were significantly lower in patients with BS than in healthy controls (Fig. 1). The absolute number of CLA^+^ Tregs (7.91/µL [interquartile range: 3.69–14.92/µL] vs. 12.56/µL [interquartile range: 8.39–18.15/µL], *P* < 0.001) was significantly lower in patients with BS than in healthy controls.

### Associations between CLA^+^ Tregs and NK cells, B cells and effector T cells

**Fig. 2:**
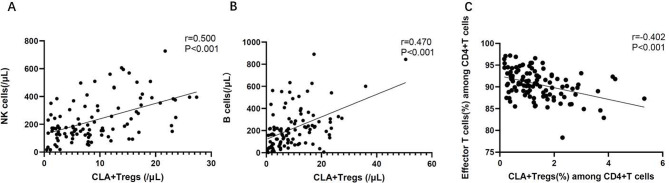
Correlation of CLA^+^ Tregs with NK cells, B cells and effector T cells. (**A**) The absolute number of CLA^+^ Tregs among CD4^+^ T cells were positively related with the absolute number of NK cells in patients with BS (*r* = 0.500, *P* < 0.001). (**B**) The absolute number of CLA^+^ Tregs among CD4^+^ T cells were positively related with the absolute number of B cells in patients with BS (*r* = 0.470, *P* < 0.001). (**C**) The proportion of CLA^+^ Tregs were negatively related with the proportion of effector T cells among CD4^+^ T cells in patients with BS (*r *= -0.402, *P* < 0.001)

The absolute number of NK cells and the absolute number of CLA^+^ Tregs (*r* = 0.500, *P* < 0.001) were positively correlated in patients with BS. The absolute number of B cells were positively associated with the absolute number of CLA^+^ Tregs (*r* = 0.470, *P* < 0.001). The proportion of CD4^+^ effector T cells among CD4^+^ T cells was negatively correlated with the proportion of CLA^+^ Tregs among CD4^+^ T cells (*r*=-0.402, *P* < 0.001) (Fig. 2).

### Associations of CLA^+^ Tregs and clinical features of BS

Patients with BS and arterial aneurysms had a lower proportion of CLA^+^ Tregs among CD4^+^ T cells than those without arterial aneurysms (0.70% [interquartile range: 0.47–0.98%] vs. 1.27% [interquartile range: 0.74–1.97%], *P* = 0.005), whereas patients with nervous system involvement had higher proportion of CLA^+^ Tregs among CD4^+^ T cells than patients without nervous system involvement (1.58% [interquartile range: 1.08–2.57%] vs. 1.09% [interquartile range: 0.56–1.75%], *P* = 0.016). The absolute number of CLA^+^ Tregs was lower in patients with arterial aneurysms than in those without (5.09/µL [interquartile range: 2.28–7.91/µL] vs. 8.25/µL [interquartile range: 3.96–15.25/µL], *P* = 0.044) and higher in patients with genital ulcers than in those without (10.45/µL [interquartile range: 5.09–15.85/µL] vs. 6.33/µL [interquartile range: 2.55–9.64/µL], *P* = 0.017). Patients with an increased ESR had a lower proportion (0.83% [interquartile range: 0.39–1.31%] vs. 1.27% [interquartile range: 0.78–1.97%], *P* = 0.013) and absolute number (5.27/µL [interquartile range: 2.18–9.23/µL] vs. 8.51/µL [interquartile range: 4.66–15.70/µL], *P* = 0.031) of CLA^+^ Tregs than those without increased ESR. (Table 2)

**Table 2:** Proportions of CLA^+^ Tregs among CD4^+^ T cells in patients with BS with different clinical features

	CLA^+^ Tregs (%) among CD4^+^ T cells				Absolute number of CLA^+^ Tregs (/µL)			
	Presence	Absence	Z	P	Presence	Absence	Z	P
Female	1.13 (0.50, 1.74)	1.22 (0.83, 1.91)	-0.711	0.477	7.78 (2.80, 15.23)	8.36 (4.97, 14.30)	-0.417	0.677
Oral ulcer	1.18 (0.68, 1.91)	0.73 (0.30, 1.76)	-0.978	0.328	7.91 (3.92, 14.92)	2.23 (1.90, 12.34)	-1.119	0.263
Genital ulcer	1.27 (0.84, 1.96)	1.00 (0.45, 1.88)	-1.763	0.078	10.45 (5.09, 15.85)	6.33 (2.55	-2.380	0.017
Pseudofolliculitis	1.12 (0.71, 1.97)	1.22 (0.56, 1.75)	-0.522	0.601	8.55 (5.15, 15.25)	7.36 (2.55, 13.30)	-1.142	0.254
Erythema nodosum	1.06 (0.45, 1.68)	1.29 (0.71, 1.97)	-1.174	0.240	8.47 (2.52, 14.42)	7.81 (4.75, 14.83)	-0.241	0.809
Uveitis	1.32 (0.65, 2.32)	1.12 (0.60, 1.78)	-0.552	0.581	7.36 (2.78, 16.97)	7.91 (3.81, 12.93)	-0.471	0.638
Arthritis	1.49 (0.56, 2.33)	1.09 (0.64, 1.73)	-1.177	0.239	10.99 (2.44, 17.07)	7.78 (4.27, 12.93)	-0.501	0.616
Gastrointestinal involvement	1.29 (0.73, 1.93)	1.12 (0.61, 1.82)	-0.440	0.660	5.88 (2.49, 14.43)	8.00 (3.69, 14.92)	-0.412	0.680
Neurological lesion	1.58 (1.08, 2.57)	1.09 (0.56, 1.75)	-2.405	0.016	9.64 (6.55, 15.85)	7.36 (2.80, 13.30)	-1.560	0.119
Fever	0.83 (0.34, 2.12)	1.20 (0.69, 1.86)	-1.216	0.224	5.27 (1.75, 11.93)	8.13 (4.26, 14.67)	-1.370	0.171
Arterial aneurysm	0.70 (0.47, 0.98)	1.27 (0.74, 1.97)	-2.777	0.005	5.09 (2.28, 7.91)	8.25 (3.96, 15.25)	-2.017	0.044
Venous thrombus	1.03 (0.76, 1.69)	1.25 (0.60, 1.93)	-0.400	0.689	9.64 (5.39, 14.92)	7.78 (2.82, 14.43)	-0.588	0.557
Positive HLA-B51	1.26 (0.73, 2.59)	1.18 (0.67, 1.91)	-0.608	0.543	13.45 (5.40, 21.91)	7.53 (4.17, 14.30)	-1.816	0.069
ESR > 20 mm/h	0.83 (0.39, 1.31)	1.27 (0.78, 1.97)	-2.490	0.013	5.27 (2.18, 9.23)	8.51 (4.66, 15.70)	-2.164	0.031
CRP > 10 mg/L	0.89 (0.40, 1.84)	1.28 (0.68, 1.87)	-1.671	0.095	5.75 (2.30, 10.36)	8.05 (3.95, 16.03)	-1.630	0.103

ESR, erythrocyte sedimentation rate. CRP, C-reactive protein

Binary logistic regression analysis showed that a decreased proportion of CLA^+^ Tregs among CD4^+^ T cells was identified as a risk factor for the development of arterial aneurysms in patients with BS (β=-1.224, SE = 0.507, *Wald* = 5.842, *P* = 0.016, odds ratio (OR) = 0.294, 95% confidence interval (CI): [0.109–0.793]). (Table 3).

**Table 3:** Logistic regression analysis of arterial aneurysms in patients with BS

Patients with arterial aneurysm						
	β	SE	Wald	P	OR	95%CI
CLA^+^Treg (%)	-1.224	0.507	5.842	0.016	0.294	(0.109, 0.793)

CLA^+^ Treg, the proportion of CLA^+^ Tregs among CD4^+^ T cells

### Changes in CLA^+^ Tregs after treatment

**Fig. 3:**
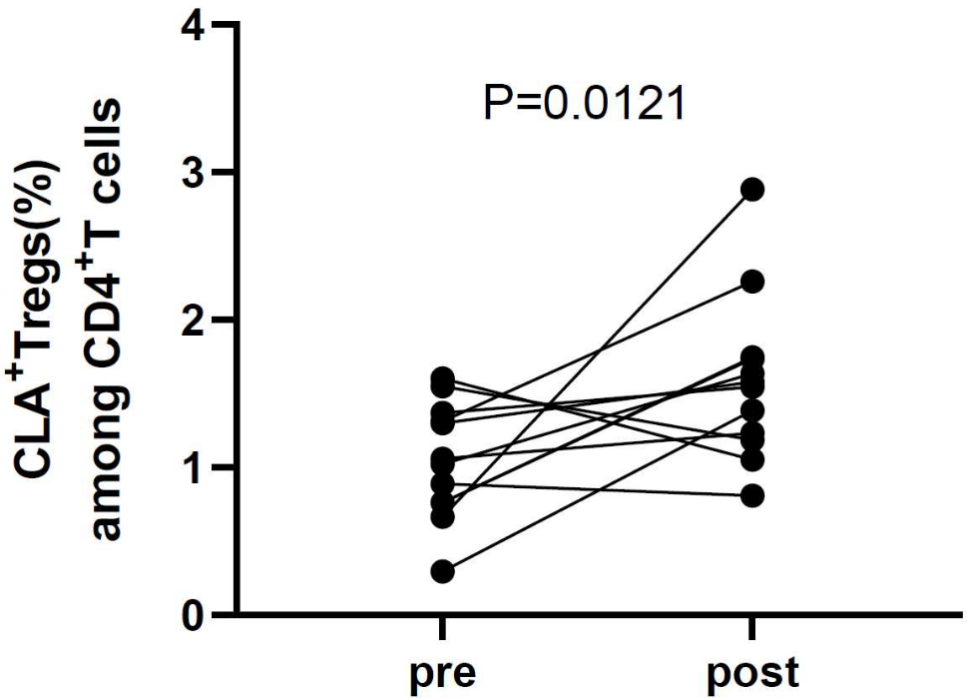
Proportion of CLA^+^ Tregs among CD4^+^ T cells before and after treatment. The BDCAF scores of patients with BS decreased (pre-treatment: 4.0 (interquartile range: 2.25–4.75); post-treatment: 2.0 (interquartile range: 1.0–3.0)). The proportion of CLA + Tregs among CD4 + T cells was significantly higher after treatment with glucocorticoids or immunosuppressants than at baseline [1.04 (interquartile range: 0.76–1.35) vs. 1.56 (interquartile range: 1.20–1.74), *P* = 0.0121]

After a median follow-up time of 11.00 months (interquartile range: 6.25–15.75 months), the BDCAF scores decreased (pre-treatment: 4.0 (interquartile range: 2.25–4.75); post-treatment: 2.0 (interquartile range: 1.0–3.0)). All 12 patients had lower BDCAF scores after follow-up and the details of 12 patients were listed in Table 4. The proportion of CLA^+^ Tregs among CD4^+^ T cells was significantly higher after treatment than at baseline [1.04 (interquartile range: 0.76–1.35) vs. 1.56 (interquartile range: 1.20–1.74), *P* = 0.0121] (Fig. 3).

**Table 4:** Details of 12 followed up patients with BS

Patient	Sex/Age (year)	Disease duration (month)	Clinical manifestations within 4 weeks before enrollment	Treatment before enrollment	Treatment after enrollment	Follow-up time (month)	BDCAF
1	Male/68	492	O, P, A, AA	GC	GC, Tocilizumab	17	4——1
2	Female/46	276	O, GU, E, A, N	GC	GC, Tocilizumab	11	5——4
3	Male/45	120	O, GU, A, T	—	GC, MMF	5	4——3
4	Male/28	120	O, E	GC	GC, MMF, HCQ	7	2——1
5	Male/29	180	O, P, A, G, N	HCQ	TAC, HCQ	11	5——3
6	Male/40	240	O, GU, U, N	GC	GC, Rituximab	4	4——3
7	Female/37	168	O, GU, E, A, G	GC	GC, Golimumab, MMF, HCQ	7	6——2
8	Female/28	28	O, G, N	Thalidomide	GC, Thalidomide, MMF, Colchicine	11	3——2
9	Female/33	204	O, GU	—	GC	21	2——1
10	Male/33	120	O, N	—	Thalidomide	12	2——1
11	Male/21	60	O, GU, E, T	GC	GC	38	4——2
12	Male/38	240	O, GU, P	—	Thalidomide	6	3——0

O, oral aphthae. GU, genital ulcers. P, pseudofolliculitis. E, erythema nodosum. A, arthritis. U, uveitis. AA, arterial aneurysm. T, thromboembolism. G, gastrointestinal involvement. N, neurological involvement. GC, glucocorticoids. TAC, tacrolimus. HCQ, hydroxychloroquine. MMF, mycophenolate morphenate

## Discussion

BS is an autoimmune disease that often has a mild onset and presents as recurrent aphthous ulcers. This condition often goes unnoticed and is not treated properly in its early stages, which may lead to severe clinical complications such as aneurysm rupture or cerebral infarction [12]. Therefore, a thorough investigation of the underlying mechanisms of BS is crucial for its early detection and effective management.

In recent years, there has been a growing interest among scholars to understand the involvement of immune cell subsets in the development of autoimmune diseases. Extensive studies have proven that various subpopulations of immune cells play a crucial role in the development of BS. Several studies have elucidated the mechanisms through which these immune cells contribute to the development and progression of BS. CLA^+^ Tregs are a subpopulation of CD4^+^ CD25^+^ Tregs. Approximately 80% of Tregs express CLA, which plays a critical role in lymphocyte homing. This unique characteristic of CLA^+^ Tregs contributes to their ability to migrate to specific tissues and exert their regulatory functions in a targeted manner. CLA^+^ Tregs can inhibit the proliferation of CD4^+^ CD25^−^ T cells by antagonizing anti-CD3, inhibiting T-cell-mediated inflammatory responses [13, 14]. In the current study, patients with elevated ESR or CRP level exhibited lower levels of CLA^+^ Tregs, suggesting that a decrease in CLA^+^ Tregs may have a significant impact on the inflammatory activity. CLAs are associated with several immune-related diseases. Leijten et al. reported that CD8^+^ CCR10^+^ T cells expressing CLAs were higher in patients with psoriatic arthritis [15], suggesting a potential role for CLA^+^ T cells in the pathogenesis of psoriatic arthritis. Similarly, Czarnowicki et al. reported increased CLA^+^ Th2 T cells and decreased CLA^+^ Th1 T cells in paediatric patients with early atopic dermatitis [16]. In the current study, the number of CLA^+^ Tregs was decreased in patients with BS, which may be attributed to an abnormal immune microenvironment that reduces the proliferation of CLA^+^ Tregs, diminishing their inhibitory effect and contributing to immune dysregulation in patients with BS. The absolute number of Tregs was negatively correlated with the ESR and CRP level in this study, suggesting that inflammation in the body is related to CLA^+^ Treg reduction. CLA^+^ Tregs may migrate to the cutaneous tissue, leading to the development of skin mucosal lesions in patients with BS and the subsequent decrease in peripheral CLA^+^ Tregs.

NK cells are cytotoxic lymphocytes that are important components of the innate immune system. Their primary function is to eliminate cells that are infected with viruses or cancerous cells [17]. The onset of BS is a multifactor-mediated process that includes both the innate and adaptive arms of the immune system, and NK cells may be involved in the pathogenesis of BS [18]. Hasan et al. reported that CD56bright CD16^−^ and CD56dim CD16^+^ NK cells were lower in the peripheral blood of patients with BS than in healthy control patients [19]. In this study, NK cells were positively correlated with CLA^+^ Tregs, which is consistent with the results of a previous study [19]. In patients with BS, CLA^+^ Tregs may interact with NK cells through cytokines, mediating immune disorders. However, the specific effects of Tregs on NK cells require further investigation. Autoantibodies are not very common in Behcet’s syndrome, which indicating humoral immunity may not play a major role in BS. Previous study showed that there was a depletion of B cells in BS compared to controls [20]. B cells were positively related with CLA^+^ Tregs in this study, suggesting decreased B cells may be associated with the reduction of CLA^+^ Tregs in patients with BS and whether different subsets of B cells can interact with CLA^+^ Tregs requires further investigation. Effector T cells play a dominant role in controlling immune and inflammatory responses in the body. CD4^+^ effector T cells include inflammatory T helper cells, such as Th1, Th2, and Th17 cells, and regulatory T cells, T follicular helper cells, and memory T cells [21]. Previous studies have shown that activated CD4^+^ effector T cells and the inflammatory cytokines they produce promote the onset of BS [22]. Shimizu et al. reported that when cultured with Th0-, Th1-, Th2-, and Th17-related cytokines and antibodies, naïve CD4^+^ T cells can upregulate genes related to these Th cells [23], indicating that increased inflammatory cytokines may promote naïve Th cell differentiation into activated effector T cells. In this study, the number of CD4^+^ effector T cells was negatively correlated with the number of CLA^+^ Tregs. The decreased number of CLA^+^ Tregs may induce an aberrant immune microenvironment in patients with BS. This hypothesis seems to be underpinned by the results for effector T cells, which increased as the number of Tregs decreased.

The results of this study suggest that CLA^+^ Tregs may influence the clinical manifestations of BS. Hsu et al. reported that CLA^+^ Tregs can affect immune tolerance in the gut [24]; however, no relationship between CLA^+^ Tregs and gastrointestinal symptoms was observed in this study. Patients with arterial aneurysms had significantly decreased proportions of CLA^+^ Tregs and logistic regression analysis showed that decreased blood CLA^+^ Treg cells was risk factors for arterial aneurysms in patients with BS. CLAs mediate the adhesion of T cells to the inflamed vascular endothelium [25]. CLA^+^ Tregs may have an immunomodulatory effect on the vascular endothelium, resulting in a higher occurrence of arterial aneurysms in individuals with lower levels of peripheral CLA^+^ Tregs. However, we also found patients with nervous lesions had higher proportion of CLA^+^ Treg. The reason why patients with BS with lower levels of peripheral CLA^+^ Tregs do not develop neurological symptoms remains unclear. The proportion of peripheral CLA^+^ Tregs among Tregs in patients with BS and nervous system lesions was similar to that in healthy controls in this study. Previous studies have reported that Tregs in the cerebrospinal fluid (CSF) may affect the inflammatory response in patients with the neuro subtype of BS [26, 27]. It was hypothesized that peripheral CLA^+^ Tregs relocate to the CSF in patients with the neuro subtype of BS, leading to low levels of peripheral CLA^+^ Tregs and influencing the neurological manifestations of BS. Future studies should measure the CLA^+^ Tregs in the CSF of patients with the neuro subtype of BS.

In the BS cohort, there is no significant disparities of the proportion and absolute number of CLA^+^ Tregs between groups. The possible reason is all cases were patients with active BS (BDCAF ≥ 1) in spite of previous medications and CLA^+^ Tregs did not differ significantly between active BS. The baseline treatment didn’t impact the CLA^+^ Tregs.

In this study, CLA^+^ Tregs were elevated in nine of 12 patients with BS whose BDCAF scores decreased after treatment, indicating that the number of CLA^+^ Tregs can increase following remission. Although CLA^+^ Tregs did not differ significantly between different active individuals with BS, the level of CLA^+^ Tregs can be increased longitudinally in patients with lower disease activity. CLA^+^ Tregs may be predictive factors for BS progression. However, CLA^+^ Tregs were decreased in 3 patients after treatment in spite of decreased BDCAF scores, which account for 25% of the total. We thought that the reason for this result is the small sample size, and expanding the sample size may get a more accurate trend.

There are limitations in this study. First, this is a retrospective study conducted in single-centre. In the future, the sample source should be expanded further. Second, we only traced the level of CLA^+^ Tregs in 12 patients. Larger studies are needed to explore the changes of CLA^+^ Treg in the disease course of BS.

## Conclusions

CLA^+^ Tregs decrease in the peripheral blood of patients with BS. CLA^+^ Tregs may influence NK cells, B cells and effector T cells in patients with BS. Patients with BS and reduced CLA^+^ Tregs may have a higher risk of co-occurring arterial aneurysms. The number of CLA^+^ Tregs can be increased after treatment for BS.

### Electronic supplementary material

Below is the link to the electronic supplementary material.

Supplementary Material 1

Supplementary Material 2

Supplementary Material 3

Supplementary Material 4

## Data Availability

The datasets used and analysed during the current study are available from the corresponding author on reasonable request.

## Abbreviations

BS: Behcet’s syndrome
CLA: cutaneous lymphocyte antigen
Treg: regulatory T cell
ESR: erythrocyte sedimentation rate
CRP: C-reactive protein
BDCAF: Behcet’s Disease Current Activity Form
GC: glucocorticoids
IS: immunosuppressants
IM: immunomodulators
