# Supplementary material for: Deficiency of peripheral CLA+ Tregs and clinical relevance in Behcet’s syndrome
Source: Arthritis Res Ther. 2024 Mar 21;26:76. doi: 10.1186/s13075-024-03306-9 (PMC10956224; doi:10.1186/s13075-024-03306-9)
Supplement: Supplementary file 1 — Supplementary Material 1 [file 13075_2024_3306_MOESM1_ESM.docx]

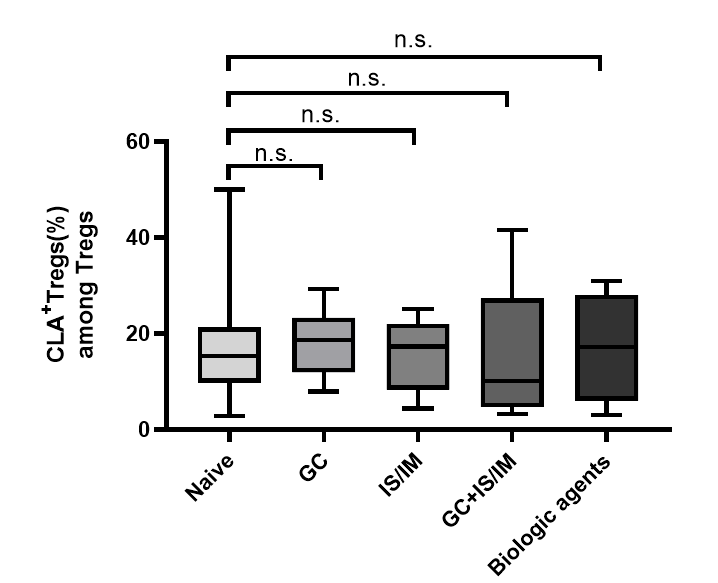


**Figure S 1. The proportion of CLA^+^ Tregs among Tregs in treatment naïve group, treated with glucocorticoids alone group, treated with immunosuppressants/immunomodulators group, treated with glucocorticoids and immunosuppressants/immunomodulators group and treated with biologic agents group. No significant difference was found between the groups. GC, glucocorticoids. IS, immunosuppressants. IM, immunomodulators.**


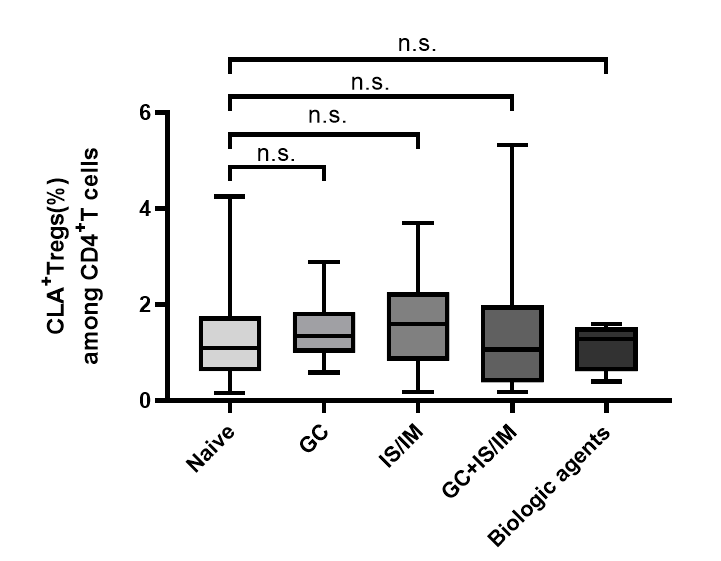


**Figure S 2. The proportion of CLA^+^ Tregs among CD4^+^ T cells in treatment naïve group, treated with GC alone group, treated with IS/IM group, treated with GC+IS/IM group and treated with biologic agents group. No significant difference was found between the groups.**


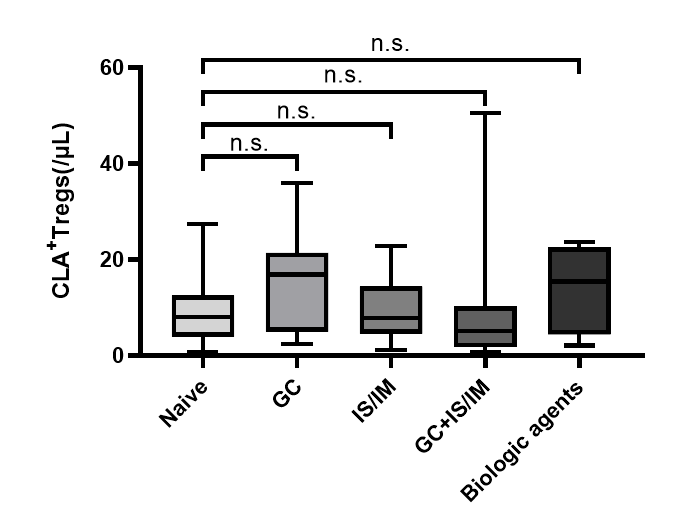


**Figure S 3. The absolute number of CLA^+^ Tregs in treatment naïve group, treated with GC alone group, treated with IS/IM group, treated with GC and IS/IM group and treated with biologic agents group.** **The absolute number of CLA^+^ Tregs in treated with GC alone group was significantly higher than that in treated with GC+IS/IM group. (P=0.0273) There was no significant difference between the other groups.**
