# Supplementary figures and images for: Deficiency of peripheral CLA+ Tregs and clinical relevance in Behcet’s syndrome
Source: Arthritis Res Ther. 2024 Mar 21;26:76. doi: 10.1186/s13075-024-03306-9 (PMC10956224; doi:10.1186/s13075-024-03306-9)

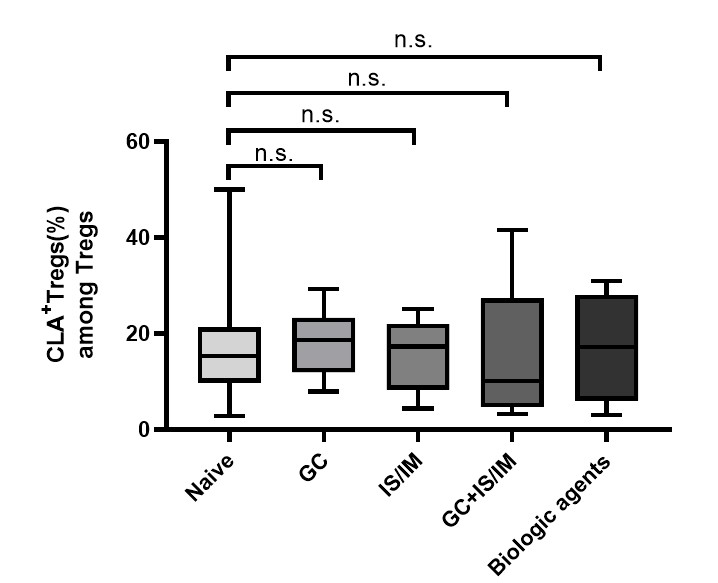

Supplement: Supplementary file 2 — Supplementary Material 2 [file 13075_2024_3306_MOESM2_ESM.jpg]

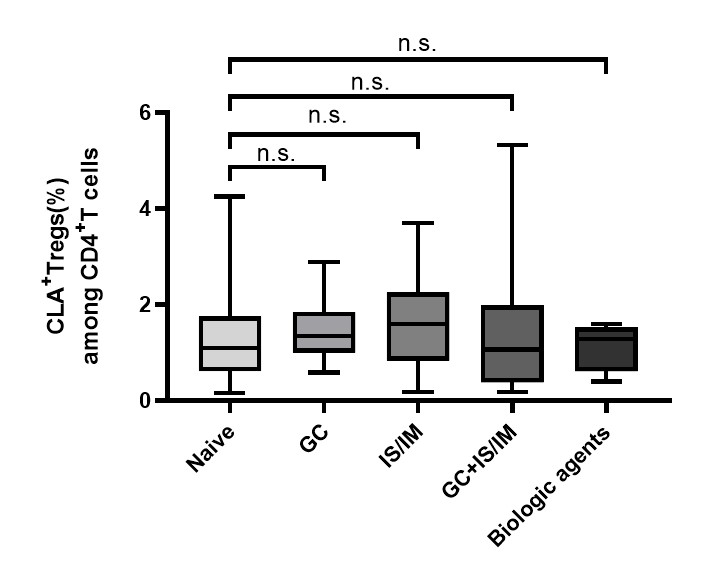

Supplement: Supplementary file 3 — Supplementary Material 3 [file 13075_2024_3306_MOESM3_ESM.jpg]

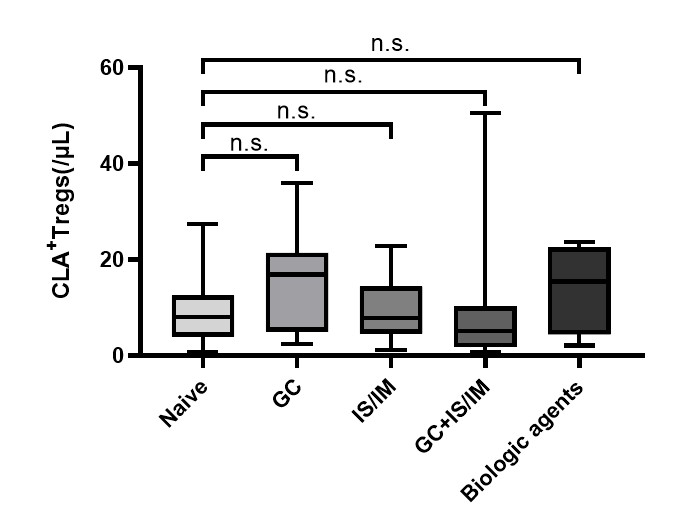

Supplement: Supplementary file 4 — Supplementary Material 4 [file 13075_2024_3306_MOESM4_ESM.jpg]
